# Supplementary material for: MetaRibo-Seq measures translation in microbiomes
Source: Nat Commun. 2020 Jun 29;11:3268. doi: 10.1038/s41467-020-17081-z (PMC7324362; doi:10.1038/s41467-020-17081-z)
Supplement: Supplementary file 10 — Supplementary Data 7 [file 41467_2020_17081_MOESM10_ESM.zip › File2/Confidence_High_Taxonomy/156414_out.krona.html]

Javascript must be enabled to view this page.

members
magnitude
magnitudeUnassigned
count
unassigned
taxon
rank

156414\_out

11

2
superkingdom
11

phylum
976
11

11
class
200643

11
1
order
171549

SRS104830\_contig\_number\_34313

10
family
171552

838
genus
3

1433844

SRS023835\_contig\_number\_contig-100\_134.83421SRS143088\_contig\_number\_14873SRS146894\_contig\_number\_16107
species
3

species
1852368

SRS014470\_contig\_number\_38511SRS049448\_contig\_number\_10373
2

370804

SRS012916\_contig\_number\_30058SRS075813\_contig\_number\_9909SRS075870\_contig\_number\_35985SRS104165\_contig\_number\_26354SRS142545\_contig\_number\_7500
species
5
